# The Natural History of Animals

**Published:** 1845-04

**Authors:** 


THE NATURAL HISTORY OF ANIMALS; being the Substance of Three Courses of Lectures delivered before the Royal Institution of Great Britain. By *Thomas Rymer Jones*, F.R.S., F.Z.S., Professor of Comparative Anatomy, in King's College, London; late Fullerian Professor of Physiology, R. I. Vol. I. pp. 362. London, Van Voorst, 1845.

THE Literature of Natural Science has, within the last few years, undergone a very remarkable change. The demand of scientific instruction for the million, and amongst the million, especially for female readers, has called forth numerous publications, of various degrees of merit indeed, but some of which, popularly as they are written, and divested as far as possible of scientific peculiarities of language and style, are not unworthy of the distinguished authors from whose condescension they have emanated. The rage for popular lectures by which the society of the present day is characterised, has, in more instances than one, occasioned the publication of "the substance" of such lectures in the form of an elementary work, on the subject on which the lectures themselves had treated; and a very cheap and expeditious mode of bookmaking has thus been adopted, by simply altering the subjective pronoun from the second to the third person, and reducing the illustrations of diagrams and realities to pretty and well-engraved wood-cuts. We confess that we see nothing to complain of in all this; on the contrary, it appears to us that it is the only way in which popular lectures can be rendered really available to the purpose of solid instruction, at least to the great majority of that class of persons who frequent them with the greatest avidity. We think we could now point out, within the small circle of our very limited acquaintance with the so-called scientific public, not a few, we will not say of ladies blue or gray, but of gentlemen, highly educated in other matters, and mixing much in scientific society, whose attendance at the popular lecture-room has been unceasing, month after month, and year after year, and yet whose information on those very matters to which they have been for so long a time and so often listening, that one would suppose they must almost have acquired the very words of their teachers by heart, brings them scarcely beyond the very threshold of science. We will not go so far as a distinguished divine, who once asked a clerical friend whether he really believed that any one person had ever derived any benefit from hearing sermons,—we will not assume so exclusive a tone as respects the comparative utility of popular lectures delivered to persons who seek only for superficial information, but we do confidently declare our belief, that no one ever became even moderately acquainted with any science whatever by listening to lectures, and especially such lectures as are ordinarily delivered in the popular theatres of our popular Institutions. The well-grounded student of Natural History or of Chemistry, frequenting the great theatre of Albemarle-street, will often come away fraught with new and beautiful facts, or with fresh arrangements of his former knowledge;—*he* will have gained real advantage. But the uninitiated, however he may be amused, will not there become a proficient, nor the superficial solidly informed.

The subject of comparative anatomy, and of zoology as associated with it, is not to be excluded from those to which we have alluded; and, in illustration of the truth of our position, we will adduce the popular Professor, the idolized of his class, male and female, wherever he holds forth—(such at least would appear to be his happy lot from the brief but pithy preface)—the author of the work which we are about to introduce to our readers. His popularity is indeed enviable. He assures us that, "the present work actually owes its origin to the flattering manner in which the lessons it contains were listened to by numerous and attentive audiences in several of the leading institutions in the Kingdom." And yet we doubt very greatly whether even our worthy Professor could put his hand upon one of his "numerous and attentive auditory" in all these various parts of the kingdom, who has, even from *his* teaching, become, we will not say a well-informed naturalist, but who has carried away from the lecture-room any well-arranged and truthful notion of the subject at all. We are aware of the great unpopularity of the opinion we are now urging. We are, indeed, greatly afraid that nothing but the absolute preservation of our incognito would save our ears, after the heretical doctrine we have broached. It is nevertheless true; nor is it, we opine, difficult to find a cause for this comparative inutility of popular lectures. However plainly and slowly the teacher may enunciate his facts or his doctrines, persons unaccustomed to the subject are wholly incapable of carrying on consecutive thought or reasoning upon it. The facts are new to them, the terms and allusions are absolutely unintelligible, and equally so whether the words by which the phenomena or facts are described or designated, rejoice in a Latin termination or an English one. Whether we talk of mollusks, molluscans, or mollusca—of mammalians or of mammalia, of pachyderms or of pachydermata, appears to us to be altogether idle. All the terms in which the descriptions are couched are novel, and therefore unintelligible to the majority of scientific lecture-hunters; and, whilst they are searching for the meaning, or endeavouring to recollect whether those terms have ever come to their ears before, the unconscious lecturer has passed on over sundry links in the chain of his reasoning, in happy conviction that his auditors have been following his course with an intelligence equal to his own. We repeat then, that we have no objection to see these fleeting and inefficient means of instruction embodied in the more tangible and available form which has in the present instance called forth the animadversions which we have thought it our duty to make.

Of the matter of which the present work is composed, and of the manner in which it has been presented to the public in its permanent dress, we will now speak.

We have no hesitation in declaring at once that this is, upon the whole, a very nice book, as far as it is as yet published; for we have now before us the first volume only, or as the author chooses to call it VOLUME ONE. Judging from the extent to which the present portion has arrived, the probability is that it will reach at least to three volumes. It is, we repeat, a very nice book; and, although containing little in it that is original—which, after all, would in most cases now-a-days be high praise—the matter it contains is judiciously chosen and well arranged. Its faults are principally those of style, and lie on the surface, and it must be reluctantly confessed that they are neither few nor slight. They are indeed so obvious and so flagrant, that it would be a degree of affectation in us, as great as that which characterises our author's style, were we to attempt to conceal or palliate them. If there be one charm in didactic writing greater than all others, one quality of style more in harmony with the pure sublimity of science, and the essential truthfulness of natnre, it is simplicity—and we are free to confess that any approach to bombast or affectation in the description of the beautiful and true and perfect works of God, is so grating to our feelings, and so repugnant to the hallowed and elevated thoughts, which the contemplation of those works is calculated to inspire, that we never meet with so derogatory an association without a painful sense of its absurdity almost amounting to disgust. To this objection our author is, we fear, in no small degree obnoxious. What less, for example, can be said of such a passage as the following, which meets us in limine?

"Is it upon the sea-shore that the student of Nature walks? Each rippling wave lays at his feet some tribute from the deep, and tells of wonders indescribable — brings corallines and painted shells, and thousand grotesque beings, samples left to show that in the sea, through all its spacious realms, life still is found—that creatures there exist more *numerously* than on the earth itself, &c." P. 2.

Again, and we select almost at random, what can possibly be made of such a jumble of metaphors as occur in the following passage? He is speaking of the coral polyps.

"Let us endeavour to picture to ourselves an extent of the bed of the ocean, spacious as these realms that we inhabit, carpeted with living plants; every blade of grass and every flower instinct with life, and all the vast expanse busily engaged in deriving from the surrounding water materials for subsistence. Let us consider that, from age to age, the wide-spreading scene is building up, by constant precipitation from the sea, a rocky territory co-extensive with itself, &c." P. 73.

First, then, the corals are living plants, blades of grass and flowers instinct with life. This metaphor is quitted abruptly, and we are introduced to an *expanse* busily engaged in deriving materials of subsistence. Then comes *a wide-spread scene* occupied in building a rocky territory as large as itself! Now, what possible force or illustration is gained by such figures as these?

Turn we the page, as our author would say, and we are presented with the following paragraph:—

"Some *accident* or *earthquake*, opens a wide chasm in the bottom of the deep; the sea itself pours through the yawning fissure, and leaps down into the fiery gulf; the imprisoned steam produced by such a dread catastrophe, putting its Titan shoulders to the vault above, heaved up the vast incumbent roof, *rocks, corals, shells and all!*"

A yawning fissure, a foaming sea, a leaping sea, imprisoned steam having Titan shoulders which it puts to the vault above!

It were too easy to multiply such examples as these, but we desist from the unpleasing and unwelcome task. We would, however, point out some of the faults arising from the same love of display and *fine* writing, such, for instance, as the incessant employment of unnecessary epithets. Thus we have in a very short space, "active polyps"—"hungry polyps"—"eating polyps"—"fishing polyps." The stomach of the Actiniæ cannot be described as capacious without the following fine phrase—"The ample folds *proclaim* its great capacity."

There are also numerous instances of carelessness which we should not mention, but in the hope that the author may be led to correct them in a future edition, which we heartily hope to see, and to avoid them in the future portions of his work. Thus we have in the description of the internal organs of the actiniæ, p. 94, the following incorrect use of the word compartments. "Between the stomach and the fleshy skin, here widely separated, are spacious cavities, divided *by compartments* from each other;" whilst, at page 97, we have the cavities themselves very properly mentioned as the compartments. As arising from the same fault of carelessness we may mention the author's expression of doubt as to the true animal nature of sponges and fungiæ compared with his calling them distinctly "forms of animal life," "such animals as these," &c., a few pages further on. Again, he declares that these animals do not possess a stomach, and afterwards adducing the sponges as an insuperable objection to the ordinary definition of an animal, that it possesses a stomach, he declares that "a stomach is all that is absolutely required. A stomach, provided it can live, is an animal."

Such instances as these are indeed of little consequence, but they evince great want of care, which might be easily avoided. On quitting this part of our task, which nothing but a sense of justice would have imposed upon us, we feel bound to say, that the faults which we have attempted to expose are far less conspicuous towards the latter part of the volume.

The remainder of our duty is indeed an agreeable one. The essential character of the book happily depends upon matters of far greater moment than mere style and taste—and as to the substance of the work, the solid material, the masonry, so to speak, we have pleasure in giving our cordial approbation, however we have been obliged to find fault with the ornamental and extrinsic adjuncts by which it is disfigured. In the first place, however, we would premise that the whole book, as far as it is as yet before the world, is a mere modification of the author's excellent work on Comparative Anatomy, "The Animal Kingdom," with certain omissions and alterations calculated to render it more acceptable "to ears polite."

The question, which our author does not profess to solve, of the point at which vegetable life ends and animal life begins, in other words, the distinction between an animal and a vegetable being, is handled as we think with considerable tact; and all the difficulties are clearly and candidly, although somewhat tediously stated. The conclusion at which he arrives, in which nothing is concluded, is that "so gradually and imperceptibly do their confines blend, that it is at present utterly out of the physiologist's power to declare exactly where vegetable existence ends, and animal life begins"—and he has the following illustration of the difficulty, which we think peculiarly happy, notwithstanding the affected manner in which it is put, and the feeble termination of the sentence. "Light and darkness are distinct from each other, and no one possessed of eyesight would be in danger of confounding night with day; yet he who looking upon the evening sky would attempt to point out precisely the line of separation between the parting day and the approaching night, *would have a difficult task to perform.*"

We do not exactly comprehend why the subject of classification is postponed to the full description of the sponges and agastric or hydroid zoophytes. However, so it has seemed fit to the Professor, and we will follow him in his progress. The system adopted is nearly that of Professor Owen; and forms perhaps the nearest approach to the truth of Nature that has hitherto been promulgated. In his Nomenclature there is however a little inconsistency. Not satisfied with adopting Latin terminations to most of his names, he has the association of an occasional English ending introduced, as it were unbidden, into the more classical society. Thus, we have in the division of the Acrita the following classes: Sponges, Polyps, Infusory Animalcules, Sterelminth*a*, Acaleph*æ*; and in the Homogargliata there are the Anneli*dans* Myriopoda, Insecta, Arachnida, Crustacea. Now, in order to be consistent, the Annelidans should be called Annelida, or else we should have Myriopods, Insects, Arachnidans, Crustaceans. These are, it is true, matters of very secondary, but still of *some* importance; and they are here indicated as fresh examples of that general carelessness in composition, to which we have seen reason before to call our author's attention.

The substance of the work is excellent. It must be a most useful book for the young zoologist, and for the general reader. Its general views, as far as they go, although we confess we do not think this department constitutes our author's strongest point, are clearly stated, and the anatomical details are given with sufficient precision. The chapter on the important subject of development is, we consider, exceedingly well done. The following illustration of the essential principles of organic development is given in a manner so graphic, as almost to deserve the praise of eloquence; and forms a very agreeable contrast to some of the passages which we have quoted as examples of a different taste.

"Every one knows how rapidly the puff-balls in our meadows are developed, growing during the course of a single night to a size that would be perfectly incredible, did not ordinary observation teach us the reality of the fact; and yet to the simple question, 'By what means is this extraordinary increase effected?' it has been quite impossible, until very recently, to give anything like a satisfactory reply. On cutting into these living masses, they are found to be quite homogeneous in their texture; the internal part consisting entirely of a spongy substance, without any vessels for the circulation of nutriment, or other complication of structure. When examined, however, with high magnifying powers, every portion of the spongy mass is seen to consist of microscopic cells, which are perpetually springing from each other, every cell producing a new cell precisely similar to itself as soon as its own growth is accomplished; so that in this way millions of millions of these vegetable cells are developed in the course of a very few hours, and by their prodigious accumulation build up a *puff-ball* measuring a foot in diameter."

Before we conclude we must not omit to notice a very material portion of this work, and one on the character of which its usefulness mainly depends—we mean the pictorial illustrations. The wood-cuts are truly first-rate. We know of nothing equal to them in the whole range of anatomical and zoological xylography. Their truth and correctness, no less than their execution, are beyond all praise. If we might select examples of peculiar beauty, we would refer to the *Lobularia,* fig. 14; the magnified view of the cells of *Madrepore,* fig. 19.; the *Rhizostoma,* fig. 63; and the interior of the *Echinus,* fig. 91. The cutting is worthy of the designs, which we presume to have been principally furnished by the talented author. We are sorry to have to make an exception to this general praise in referring to the wretched unintelligible figure of *Velella,* fig. 68, which, instead of being light and transparent, is as solid as iron, and almost as opake.

We conclude with a cordial recommendation of the work; and with the hope that the animadversions which it has been our unpleasant duty to make, may induce the author "projicere ampullas et sesquipedalia verba," and to avoid also the careless and, we were going to say, lazy habit that has so materially deteriorated this otherwise excellent book.

